# Evolution of Seed-Soluble and Insoluble Tannins during Grape Berry Maturation

**DOI:** 10.3390/molecules28073050

**Published:** 2023-03-29

**Authors:** Jingjing Wang, Xuechen Yao, Nongyu Xia, Qi Sun, Changqing Duan, Qiuhong Pan

**Affiliations:** 1 Center for Viticulture & Enology, College of Food Science and Nutritional Engineering, China Agricultural University, Beijing 100083, China; 2 Key Laboratory of Viticulture and Enology, Ministry of Agricultural and Rural Affairs, Beijing 100083, China

**Keywords:** soluble tannin, insoluble tannin, flavan-3-ol unit, mean degree of polymerization, grape seeds

## Abstract

Condensed tannins (CT) in wine are derived from the seeds and skins of grapes, and their composition and content contribute to the bitterness/astringency characteristics and ageing potential of the wine. Global warming has accelerated the ripening process of grape berries, making them out of sync with seed ripening. To understand the influence of berry ripening on the seed CT composition and content, we analyzed the changes in the soluble and insoluble CT in the seeds of ‘Cabernet Sauvignon’ grapes from two vineyards over two years. The results showed that the seed-soluble CT presented a slight downward trend in fluctuation during grape berry development, while the insoluble CT increased continuously before the véraison and remained at a high level afterwards. Relatively speaking, a lower sugar increment in developing grape berries favored the conversion of seed CT towards a higher degree of polymerization. The terminal unit of soluble CT was dominated by epigallocatechin gallate, the content of which decreased as the seeds matured. It is suggested that the seeds should be fully matured to reduce this bitter component in tannins. This study provides a reference for us to control the grape ripening process and produce high-quality grapes for wine making.

## 1. Introduction

Tannins are phenolic compounds found in grapes and wine. Tannins in wine are divided into two types: hydrolyzed tannins and condensed tannins. Hydrolyzed tannins are mostly gallotannins from grape berries and ellagitannins from oak barrels [1], while condensed tannins are derived from the grapes and are the main contributors of wine astringency. Condensed tannins (CT), also known as proanthocyanidins, are polymers of flavan-3-ols. CT is a class of important secondary metabolites that not only protect the fruits from animals and pathogens [2], but also have potential antioxidant activity and beneficial pharmacological properties, such as anti-obesity, anti-diabetes, anti-neurodegeneration, anti-cancer, and cardio protection [3,4]. Meanwhile, CT is also an important quality component. In wine, CT largely determines the intensity and characteristics of astringency and bitterness, color stabilization, and ageing potential [5,6]. The astringency intensity of wine is positively correlated with the CT content, which depends on the extraction and solubility of CT from grapes [7,8]. Based on the solubility, CT are generally divided into soluble and insoluble CT [9]. Soluble CT in grapes can be immersed in wine, whereas insoluble CT have high mean degrees of polymerization (mDP) and are cross-linked with cell wall proteins, polysaccharides, and other cellular components, resulting in a decrease in the solubility [10]. As the CT polymerization degree increases, the maximum intensity (Imax) of astringency increases, while the Imax and total duration (Ttot) of bitterness decrease [6,11,12]. In addition, the sensory characteristics of CT in wine are related to the composition of the CT units. Studies have shown that (−)-epicatechin has a higher Imax and Ttot than (+)-catechin in terms of bitterness, and the intensity of astringency and bitterness increases with the increase in the gallic esterification degree of the C-ring and the hydroxyl number of the B-ring in the CT component units. Among the flavan-3-ol monomers commonly found in grape berries, (−)-epigallocatechin gallate is considered to be the most astringent flavan-3-ol monomer in grapes [11,13,14]. Overall, the content and subunit composition of CT are important elements influencing the wine astringency.

CT are mainly found in grape seeds and skins, with a higher concentration in seeds. It has been estimated that about 60% of the CT in wine come from seeds and the rest from skins [15]. Therefore, the composition and accumulation of seed CT during grape berry development could have a significant influence on the quality of the wines produced.

There are still some conflicting results regarding the pattern of CT accumulation in seeds during grape ripening. In general, it is agreed that CT synthesis starts at the flowering stage of grapevines and reaches a peak at véraison (which represents the onset of berry ripening and is characterized by important biochemical and physiological changes such as softening and coloring.), followed by a decline [16]. The decrease in CT content after véraison is speculated to be due to the formation of oxidative cross-linking products, which strongly bind with the proteins and polysaccharides of cell walls, thus reducing the extractable rate of CT [17]. However, some studies also indicate no significant evolution of CT content in the seeds during the ripening stage of grapes [18,19,20]. The different conclusions obtained may be related to the grape varieties and the solvents or methods used for CT extraction, as well as the influence of abiotic factors such as solar radiation, temperature, water availability, altitude, ripening process, and cultivation practices [5]. Further research is needed to investigate the accumulation pattern of soluble and insoluble CT in grape seeds in wine-growing regions with specific climates, with the aim of providing a reference basis for selecting appropriate management measures and predicting the optimal commercial harvest time.

The eastern foothills of Helan Mountain are located between 105°45′–106°27′ E and 37°43′–39°23′ N and are one of the youngest wine-growing areas in China. This region belongs to a continental arid and semi-arid climate with the accumulated temperature (≥10 °C) of 3400~3800 °C in the grape growing season, sunshine at 1700~2000 h, and the precipitation of 150~240 mm. In addition, there is a large daily temperature difference of 12~15 °C. These climatic characteristics make the sugar accumulation of the grape berry in this region too fast, and it is very common that the seed maturity lags behind the sugar accumulation of the grape berry in the grape growing season. Typically, wines made from such grapes have an alcohol content of 13.5–14% (*v*/*v*) when the natural sugar content of the berry reaches about 25 ^o^Brix, which is close to the upper limit for the survival of most alcohol-tolerant yeasts. In addition, a titratable acidity from 6.5 to 8.5 g/L is considered to be the optimum concentration for the production of well-balanced wines [21]. In order to obtain better seed tannins, growers have to delay the harvesting. As a result, the soluble solids at harvest even reach 26–28 ^o^Brix, while the titratable acid drops to 5–6 g/L. High soluble sugars and low acidity in grape berries can make subsequent alcoholic and malolactic fermentation difficult, resulting in wines with high volatile acidity and alcohol content and poor balance [22]. High alcohol levels also inhibit the volatilization of wine aromas [23]. To help winemakers to determine the appropriate time to harvest, it is important to understand the effect of berry sugar increment on the composition and content of seed tannins.

The complexity of the CT structure and the susceptibility of seed CT to oxidative cross-linking reactions make it difficult to quantify CT in seeds [24]. In the past, colorimetric analysis has also been routinely used to determine seed CT. However, both colorimetric and chromatographic methods are time-consuming for the rapid determination of appropriate harvest dates of wine grape berries in practical use. Ristic and Iland [25] developed a progressive seed coat color chart to estimate the seed ripening. Avila and his colleagues [26] analyzed the relationship between seed coat color changes and CT oxidation, and proposed the possibility of using simple color measures to predict seed maturity. More recently, grape seed textural characteristics (width, area, transverse and vertical diameter, etc.) and seed coat color have been described using hyperspectral imaging analysis techniques to effectively discriminate seed maturity stages [27].

In this study, we investigated the accumulation pattern and composition of soluble and insoluble CT in seeds during the ripening process of ‘Cabernet Sauvignon’ grapes grown at the eastern foothills of the Helan Mountains in Ningxia, China over two vintages. The correlation between CT content and seed color parameters was analyzed. This research aims to provide a basis for determining the optimal commercial harvesting period of wineries and the design of personalized quality wine production processes.

## 2. Results

### 2.1. Comparison of Grape Berry Ripening Process

**Figure 1:**
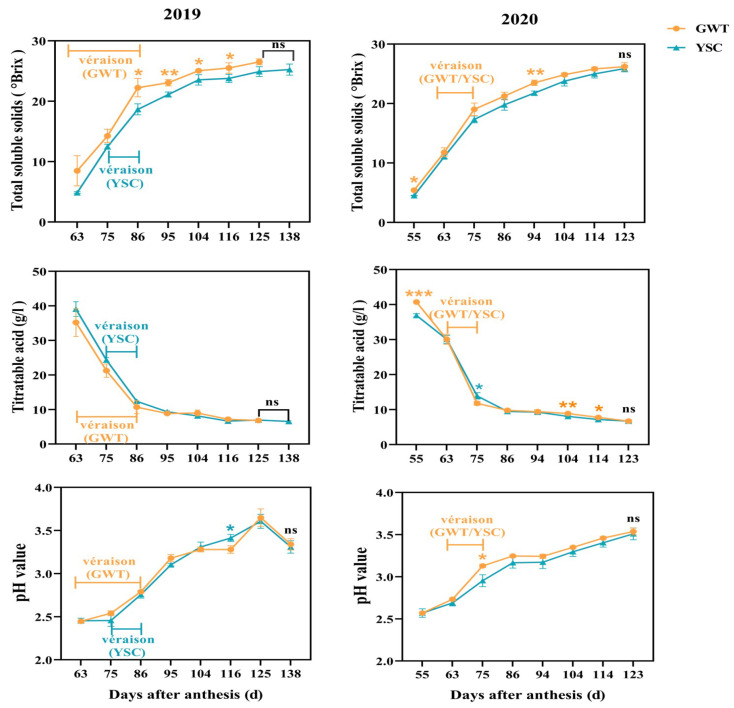
Changes in total soluble solids, titratable acid content, and pH value during ripening of ‘Cabernet Sauvignon’ grapes from two vineyards in 2019–2020. In 2019, ‘Cabernet Sauvignon’ grapes from the GWT entered the véraison stage at 63 DAA, full coloring stage at 86 DAA, and commercial harvesting stage at 125 DAA. The grapes from the YSC started coloration at 75 DAA, full coloring at 86 DAA, and commercial harvesting at 138 DAA. In the 2020 vintage, grapes from both vineyards started véraison at 63 DAA, full coloring at 75 DAA and commercial harvesting at 123 DAA/The asterisk indicates that there is a statistical difference between the 2 vineyards in the same period at the 0.05 level: *, *p* < 0.05; **, *p* < 0.01; ***, *p* < 0.001; ns, not significant.

The comparison of changes in total soluble solids and titratable acid revealed differences in the ripening process of the ‘Cabernet Sauvignon’ grapes from the Great Wall terroir (GWT) and the Yuanshi Chateau (YSC) vineyards. The total soluble solids of the GWT grapes were higher than that of the YSC grapes at the same stage for 2 consecutive years, with a significant difference from 86 to 116 days after anthesis (DAA) for the 2019 vintage and from 55 to 94 DAA for the 2020 vintage. As noted in Figure 1, the submitted records indicated that the grapes from GWT entered véraison 12 days earlier than YSC in 2019, reaching commercial harvest at 125 DAA, while the grapes from YSC were commercially harvested at 138 DAA. It was suggested that the ripening rate of GWT grapes was relatively faster than that of YSC in the 2019 vintage. In contrast, there was a similar ripening process of the 2 vineyards in 2020, with both reaching a commercial harvest at 123 DAA. The grapes from the two vineyards did not show any difference in pH and titratable acid content at harvest (Figure 1).

### 2.2. Change of Seed Coat Color

**Figure 2:**
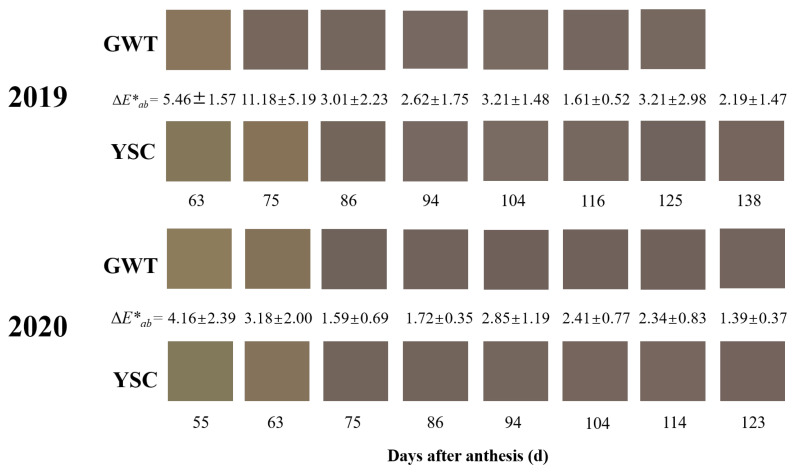
Color simulation plots of GWT and YSC grape seeds. $\Delta E^{*}{}_{ab}$ represents the color difference value between the seeds from the two vineyards in the same period.

Figure 2 shows the simulation plots of seed coat color for the 2019 and 2020 vintages at the same developmental stage. Intuitively, it could be seen that the GWT seeds in 2019 had already turned a lighter shade of brown at 63 DAA, while the YSC seeds remained green at this time. At 75 DAA, the browning of the GWT seeds was complete, but the YSC seed coats were still light brown. The difference in appearance (ΔE*_ab_) of the seed coat color between the 2 vineyards was greater than 3 a.u. in the first 2 periods, representing a macroscopically discernible color difference [28]. Similarly, in 2020, the discernible difference in seed coat color between the GWT and YSC seeds was presented at 55 DAA and 63 DAA, with a color difference of 4.16 ± 2.39 a.u. and 3.18 ± 2.00 a.u. The results indicated that the browning of the GWT seed coat was earlier than that of the YSC seeds, which is related to the early onset of ripening of ‘Cabernet Sauvignon’ grapes in the GWT.

### 2.3. Comparison of Soluble and Insoluble CT Contents

**Figure 3:**
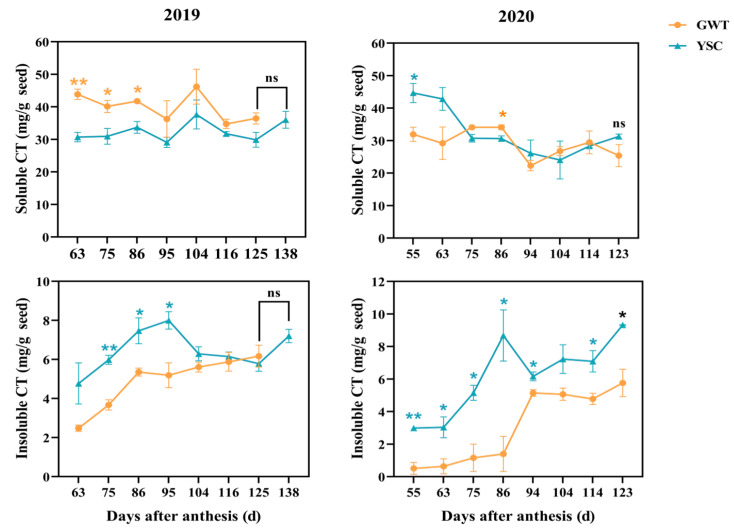
Variation of soluble and insoluble CT content in GWT and YSC seeds in 2019 and 2020. The asterisk indicates that there is a statistical difference between the 2 vineyards in the same period at the 0.05 level:*, *p* < 0.05; **, *p* < 0.01; ns, not significant.

The changes in soluble and insoluble CT content in seeds during the ripening of ‘Cabernet Sauvignon’ grapes were investigated (Figure 3). Throughout the grape berry ripening in both years, the soluble CT content of the seeds showed an overall declining trend. For the comparison between the 2 vineyards, the soluble CT content of GWT seeds in 2019 was significantly higher compared to the YSC seeds from 63 to 86 DAA, which should be related to the earlier ripening start of GWT grapes, and in 2020, the soluble CT content did not show a difference between the seeds from the GWT and YSC grapes except at 55 DAA and 86 DAA. A comparison of the variation patterns between the two years within the same vineyard showed a significant difference. A two-way ANOVA indicated that soluble CT accumulation in the seeds varied markedly with the vintage, but not with the plots. There was also an interaction between the plot and the year on seed-soluble CT accumulation (Table 1).

The insoluble CT content in the seeds showed an initial upward trend during berry ripening and finally stabilized at a certain level. It was found that the insoluble CT content in the seeds varied significantly between the two vineyards, with the YSC seeds showing a higher level compared to the GWT in both years. Moreover, the insoluble CT of the seeds from both vineyards showed a rapidly increasing trend from the véraison stage (63 and 75 DAA in 2019 and 63 DAA in 2020), which seemed to be related to the decrease in soluble CT during this period. It was assumed that the soluble CT were further polymerized to insoluble CT with ripening, as reported previously [29,30].

At harvest, there were no significant differences in the content of soluble and insoluble CT between the two vineyards, except that the seeds from YSC had a higher content of insoluble CT (Figure 3).

### 2.4. Comparison of Soluble and Insoluble CT Flavan-3-ol Units

**Figure 4:**
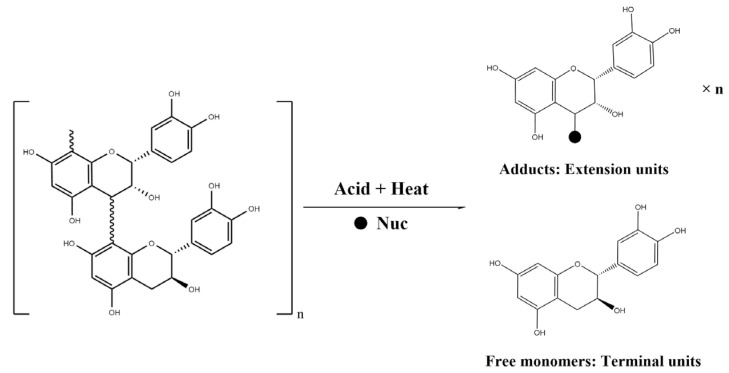
Nucleophilic reagent hydrolysis of condensed tannins under heated acid condition. Nuc: abbreviation for nucleophilic reagent.

**Figure 5:**
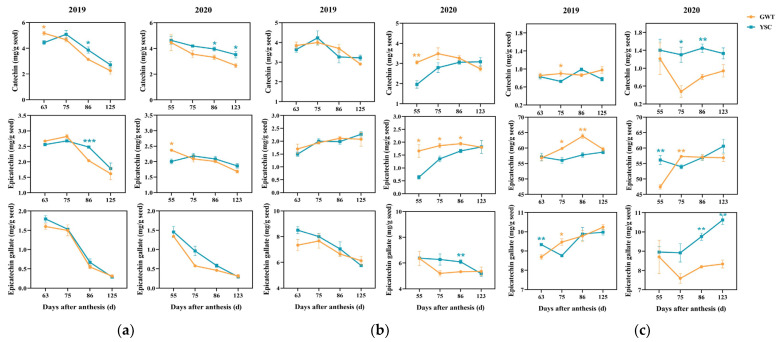
Changes in the content of free flavan-3-ol monomers (**a**), terminal units (**b**), and extension units (**c**) in soluble CT of GWT and YSC seeds. The asterisk indicates that there is a statistical difference between the 2 vineyards in the same period at the 0.05 level: *, *p* < 0.05; **, *p* < 0.01; ***, *p* < 0.001.

CT can be hydrolyzed by nucleophilic reagents in an acidic environment, and the flavan bond is broken, releasing the terminal units in the form of flavan-3-ol monomer and the extension units in the form of carbon positive ions (Figure 4) [31]. To understand the changes in free flavan-3-ol monomers and the constitutional units of soluble and insoluble CT, the seeds from four-time points were selected for further analysis (Figure 5). The changes in the degree of polymerization (mDP) of soluble and insoluble CT during berry ripening were also evaluated. In this study, three flavan-3-ol types were identified in the seeds: (+)-catechin, (−)-epicatechin and (−)-epicatechin-3-gallate.

#### 2.4.1. Free Flavan-3-ol Monomers in the Seeds

Free flavan-3-ol monomers refer to compounds that have not yet participated in the condensation reaction of tannins. In the present study, free flavan-3-ol monomers mainly included catechin, epicatechin, and epicatechin gallate, and their levels all showed a decreasing trend with ripening (Figure 5a). Comparing the difference between the 2 vineyards, it was found that only at 63 and 86 DAA in 2019 and 86 and 123 DAA in 2020 were significant differences in the catechin content. For epicatechin, the difference between the two vineyards was only found at 86 DAA in 2019 and at 55 DAA in 2020. For epicatechin gallate, there was no difference between the vineyards (Figure 5a).

#### 2.4.2. Flavan-3-ol Composition Units of Seed-Soluble CT

For the terminal units of seed-soluble CT (Figure 5b), the highest content was found in epicatechin gallate, followed by catechin and epicatechin. The content of epicatechin gallate decreased significantly with ripening, while the other two units changed slightly. In 2019, the three flavan-3-ol units did not show any differences between the two vineyards. However, in 2020, the difference in the epicatechin gallate content between the 2 vineyards appeared at 86 DAA, and the content in the YSC grape seeds of grapes was higher than that in the GWT seeds. The catechin content in GWT was significantly higher than that in YSC at 55 DAA in 2020, and the epicatechin content was significantly higher in GWT at most periods.

For the extension units of seed-soluble CT (Figure 5c), epicatechin accounted for an absolutely high proportion and its content was much higher than the other two components. Epicatechin and epicatechin gallate units showed an overall increasing trend with ripening, while catechin showed little change. The catechin in the GWT seeds was lower at most stages of 2020, and the epicatechin in GWT, except for 75 DAA, was higher or similar to that of YSC. The epicatechin gallate unit varied somewhat between the two vineyards, especially in 2020 when the accumulation of epicatechin gallate in the YSC seeds was significantly higher than that of GWT.

#### 2.4.3. Flavan-3-ol Composition Units of Insoluble CT

**Figure 6:**
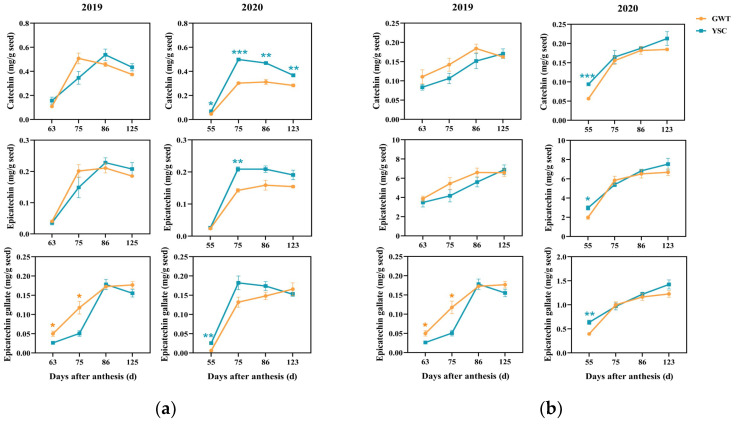
Changes in the content of terminal units (**a**) and extension units (**b**) in insoluble CT of GWT and YSC seeds. The asterisk indicates that there is a statistical difference between the two vineyards in the same period at the 0.05 level: *, *p* < 0.05; **, *p* < 0.01; ***, *p* < 0.001.

For the terminal units of insoluble CT in the seeds (Figure 6a), three components were at almost similar levels. The catechin content reached a peak at the full coloring of the grape berry (86 DAA in 2019 and 75 DAA in 2020), and then slightly decreased and remained at a certain level. The GWT grapes also had a faster ripening process in 2019, and the peak content appeared at 75 DAA. In addition, we noticed that the accumulation of terminal unit catechin in the YSC seeds of 2020 was significantly higher than that of GWT in the four periods detected, and the epicatechin content was also higher at 75 DAA. However, both catechin and epicatechin did not show any differences between the vineyards in 2019. The content of epicatechin gallate was the lowest among the compositional units, and the difference between the vineyards also changed with the vintage.

The three extension units of insoluble CT all showed an increasing trend with ripening (Figure 6b). Among them, the epicatechin content was much higher than the other two components. In 2019, the content of catechin and epicatechin did not show a difference between the vineyards, and the content of epicatechin gallate was higher at 63 and 75 DAA in the GWT grape seeds than that in the YSC seeds. In 2020, except for a higher content in YSC seeds at 55 DAA, the 3 flavan-3-ol units did not show any differences between the vineyards at the other stages. In addition, the epicatechin gallate content was found to be significantly higher in 2020 than that in 2019.

#### 2.4.4. Mean Degree of Polymerization of Soluble and Insoluble CT

**Figure 7:**
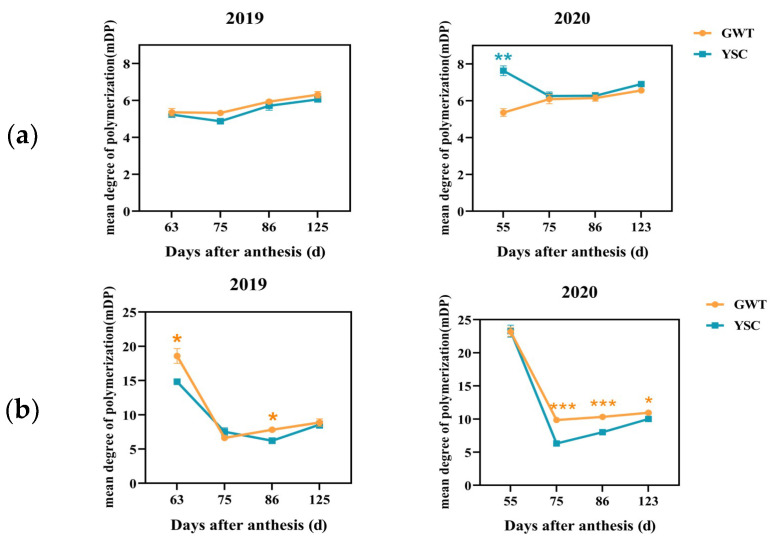
Changes in the mean degree polymerization of soluble (**a**) and insoluble CT (**b**) in the GWT and YSC seeds. The asterisk indicates that there is a statistical difference between the 2 vineyards in the same period at the 0.05 level: *, *p* < 0.05; **, *p* < 0.01; ***, *p* < 0.001.

The change in the mean degree of polymerization (mDP) of seed-soluble CT generally showed a moderately increasing trend (Figure 7a). There was no significant difference in the mDP values of seed-soluble CT between the 2 vineyards, except at 55 DAA in 2020. The mDP ranged from 4.87 to 6.31 for the 2019 seeds, while the mDP was slightly higher, ranging from 5.36 to 7.63 for the 2020 seeds.

The mDP of insoluble CT was significantly higher than that of soluble CT, ranging from 6.24 to 23.36 (Figure 7). The mDP of insoluble CT showed similar variation trends in 2019 and 2020, with a rapid decrease up to 75 DAA and a slight increase thereafter. The mDP of insoluble CT of GWT seeds was significantly higher than that of YSC at 63 and 86 DAA in 2019 and at 3 periods in 2020 (75 DAA, 86 DAA, and 123 DAA), respectively. Furthermore, the mDP of insoluble CT was higher overall in 2020 than in 2019, which was similar to the presentation of soluble CTs’ mDP.

### 2.5. Correlation Analysis between Seed CT and Ripening Parameter

**Figure 8:**
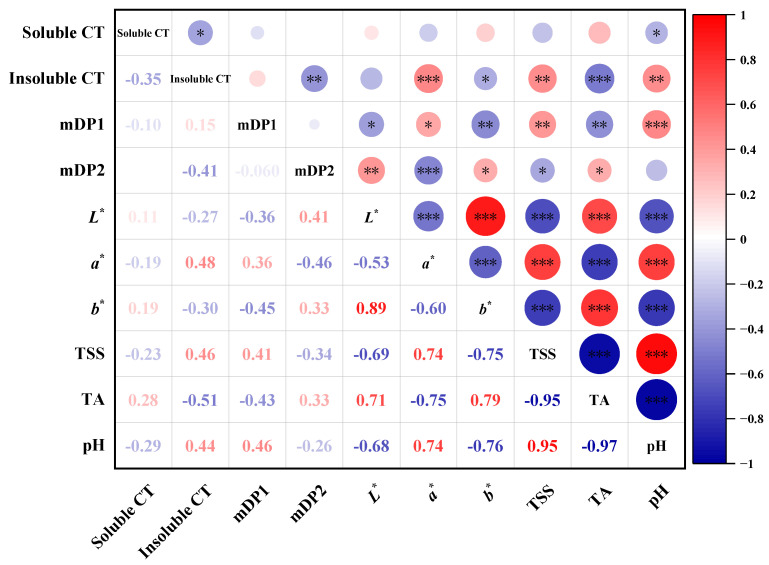
Correlation of seed tannin content with seed coat color index and berry ripening index. mDP1 and mDP2 represent the mean polymerization degree of soluble and insoluble CT, respectively. TSS represents the total soluble solid and TA is the titratable acidity. L* represents the lightness indicator (L* = 0 for black, L* = 100 for white). a* and b* are two color indicators (a* > 0 for red, a* < 0 for green; b* > 0 for yellow, b* < 0 for blue). *** represents a significant correlation at *p* < 0.001, ** represents a significant correlation at *p* < 0.01, and * represent a significant correlations at *p* < 0.05.

In order to analyze the effects of the ripening process on the composition and content of seed CT, the correlation between CT and various ripening parameters mentioned above was analyzed. As shown in Figure 8, the soluble CT content was not significantly correlated with the seed coat color index, and the mDP of soluble CT (expressed as mDP1) showed a weak positive correlation with the a* value of the seed coat color (0.36) and a weak negative correlation with the b* value (−0.45). This indicated that an increase in the soluble CT polymerization degree would cause seed coat browning, a sign of seed maturity. In addition, mDP1 was positively correlated with the total soluble solids and pH values, and negatively correlated with the titratable acid content. The above results all suggest that the polymerization degree of seed-soluble CT increases with fruit ripening.

For seed insoluble CT, their content was positively correlated with the a* value of the seed coat color, the total soluble solids content, and the pH value. In contrast, the insoluble CT content was negatively correlated with the b* value and the titratable acid content (Figure 8). The results clearly show that the insoluble CT in the seed increase continuously with the browning of the seed coat as the berries ripen. This means that the color of the seed coat may reflect to some extent the content of insoluble CT.

## 3. Discussion

Tannins have a significant influence on wine astringency, color stability, and ageing potential [2]. This study found that, during the ripening process of ‘Cabernet Sauvignon’ grapes, the content of seed-soluble CT and flavan-3-ol monomers fluctuated and decreased slightly, whereas the content of insoluble CT increased continuously until véraison and decreased slightly but remained at a high level after the full coloring. Similar variation trends have been reported previously, where researchers have found a decrease in soluble tannin content in seeds with grape ripening [25,32,33]. This is thought to be related to tannin oxidation and the gradual formation of seed coats. During seed ripening, seeds undergo oxidative browning, drying, and water loss, which are accompanied by the oxidation of CT. At the same time, previously synthesized CT can be oxidatively cross-linked with macromolecules in the cell wall to form insoluble CT [29]. In addition, the CT oxidation process allows for the continuous polymerization of flavan-3-ol monomers and produces non-homogeneous products (e.g., the heterocyclic C-ring binding B-ring), which reduce the hydroxyl binding sites necessary for protein precipitation, thereby reducing the bitterness and astringency of seed CT and affecting the sensory quality of the wine [34,35]. In this study, the grapes from the GWT vineyard showed a faster ripening process; correspondingly, the GWT seeds had a lower content of insoluble CT. Furthermore, the insoluble CT content was positively correlated with the seed coat browning and berry ripening index. The overall result suggests that slowing down the berry ripening process facilitates tannin polymerization and seed maturation. This result is consistent with our previous research [30]. It is known that wine bitterness is mainly contributed to by monomeric flavan-3-ols and low polymerization degrees of CT, whereas astringency is mainly contributed to by high polymerization CT [36,37]. Studies have demonstrated that high degrees of polymerization CT (mDP > 20) extracted from both apples and grapes are readily soluble in wine-like hydroalcoholic solution systems and are highly astringent [14,38]. However, the present study lacks sufficient data to illustrate how much soluble and insoluble CT from seeds can enter the wine during the winemaking process. Nevertheless, it is clear that the vineyard practices that slow down the berry ripening process are beneficial in reducing wine bitterness and improving astringency.

It is generally believed that soluble CT are relatively easy to extract from seeds and transfer to wine. This study shows that the terminal units of soluble CT in seeds were mainly epicatechin gallate and their content decreased during ripening, whereas the extension unit was mainly epicatechin and its content increased steadily. This suggests that the galloylation proportions of soluble CT gradually decrease as the seeds ripen, which would ultimately affect the sensory quality of the wine. Previous researchers have indicated that both the difference in solubility and the spatial conformation of tannins can significantly influence the ability of tannins to bind to oral proteins, thus affecting the bitter and astringent flavors [39,40,41]. In general, CT with more galloylation have a greater astringency because there are more aromatic and hydroxyl groups introduced by the gallate, which promote the non-covalent binding of the CT to oral salivary proteins [42,43]. However, this astringency is perceived as “unpleasant”, slightly dry, rough, and grainy [44]. Therefore, seed-soluble CT with lower galloylation are required for the production of high-quality wine.

Global warming has accelerated the fruit ripening process in recent years [45], and the phenomenon of fruit sugar accumulation and phenolic accumulation, as well as seed ripening asynchrony, has become more apparent during harvest [46,47]. In view of this, winemakers consider the phenolic maturity of grapes, particularly seed maturity, as a crucial indicator for determining the ideal harvest date [36]. Currently, there are two methods for assessing seed maturity. One is the sensory tasting method, which involves the winemaker’s subjective evaluation [34]; and the other is the determination method of color, texture, and other parameters, which involves establishing the correlation between the phenolic content of the seed and its physical parameters [42,48]. With the development of technology, the acquisition of seed coat color and physical parameters such as hardness and length have become more accurate. Investigating the relationships between seed coat color indicators and the evolution of phenolic compounds in seeds have also become an important method for determining the level of seed maturity. In this study, the correlation analysis revealed that the mDP values of both soluble CT and the content of insoluble CT were significantly positively correlated with the seed coat a* value and the total soluble solids’ content, but were significantly negatively correlated with the b* value, indicating that seed tannins changed to a higher degree of polymerization with fruit ripening, and this change was closely related to the oxidative browning of the seed coat. It is suggested that the seed coat color parameters can be used to predict the polymerization of CT during seed development to a certain extent, which is beneficial to provide a reference basis for winemaking.

## 4. Materials and Methods

### 4.1. Information of Vineyards and Sampling

This experiment was carried out in 2 vineyards planted with *Vitis vinifera* L. cv. Cabernet Sauvignon, owned by the Great Wall terroir (GWT, 105.95° E, 38.39° N) and the Yuanshi Chateau (YSC, 106.04° E, 38.58° N), both located at the eastern foothills of the Helan Mountains. Basic information about the vineyards is as follows: the Great Wall terroir experimental vineyard covers approximately 240 hectares of alkaline, gravelly soil; the vines were self-rooted seedlings planted in 2012 and trained to uniformly modified vertical shoot positioning (M-VSP) in north-south rows at 0.8 m × 4 m spacing; the vineyard of the Yuanshi Chateau vineyard covers an area of 266 hectares with sandy loam and alkaline soils; the vines were self-rooted seedlings planted with M-VSP in 2013; and the planting orientation was north-south, with a vine and row spacing of 1.2 m × 4 m.

This study was conducted in the 2019 and 2020 vintages, with berries collected at 10-day intervals from the beginning of 25 July 2019 and 19 July 2020, respectively, until commercial harvest (Table 2). It should be noted that the grapes in both vineyards flowered on 23 May 2019 and 25 May 2020, respectively. According to the phenological data recorded by the growers over the last 10 years, the date when the vines in the 2 vineyards entered the flowering period was very close, which may be because the vines in this region must all be buried in the soil to avoid dying in the dry and cold winter until the air temperature exceeded 10 ℃ in the coming spring. In the different vineyards of this wine region, the date of digging was very close, which caused the vines to flower at almost the same time.

For grape sampling, three biological plots were set up in each vineyard using a randomized block design. Each biological plot consisted of 3 rows of vines, with approximately 40 m of spacing between the biological plots. In each row, ten vines with the same growth potential were selected for berry collection. A total of 300 berries were collected from 50 clusters with 6 berries per cluster at each sampling before 10 a.m. After collection, the samples were immediately placed in ice boxes and transported to the physicochemical laboratory within 1 h. Of these, 50 fresh berries were used for physicochemical parameters, while the remainder were rapidly frozen in liquid nitrogen and stored at −80 °C for seed tannin analysis.

### 4.2. Methods

#### 4.2.1. Determination of Total Soluble Solids, Titratable Acid, and pH in Grape Juice

In total, 50 grape berries were previously deseeded and crushed to obtain the juice, centrifuged at 5000 rpm, and the clarified juice was collected. The total soluble solids content was determined using a digital hand-held refractometer (PAL-1, Atago, Tokyo, Japan) and the result was expressed in °Brix. The pH of the juice was measured using a pH meter (Sartorius PB-10, Göttingen, Germany). The titratable acidity was titrated with pre-calibrated NaOH (0.05 mol/L), and the result was expressed as the tartaric acid equivalent (g/L).

#### 4.2.2. Seed Coat Color Determination

The CIELAB space was used to assess the color parameters of the seed coat: L*, a*, and b*, where L* is the lightness indicator (L* = 0 for black, L* = 100 for white) and a* and b* are two color indicators (a* > 0 for red, a* < 0 for green; b* > 0 for yellow, b* < 0 for blue) [49]. Furthermore, the color difference between the seeds from the two vineyards in the same period was calculated using the Equation (1).

(1) $$\Delta E^{*}{}_{ab}=\sqrt[{2}]{{\left(\Delta L^{*}\right)}^{2}+{\left(\Delta a^{*}\right)}^{2}+{\left(\Delta b^{*}\right)}^{2}}$$

The seed coat color was measured using the CM-3700d spectrophotometer (KONICA MINOLTA, Osaka, Japan). The chronometer settings were D65 light source, 10° visual observation angle and MAV (8 mm) sample measuring aperture. A zero calibration was performed prior to each sample measurement and a white calibration was performed using a typical white calibration plate. Ten seeds were randomly chosen for each biological replicate. Each biological replicate was measured three times, and all of the data were averaged.

#### 4.2.3. Extraction and Determination of Soluble and Insoluble CT

The extraction, determination, and compositional unit analysis of soluble and insoluble CT in the seeds were essentially based on the method described in our recent publication [30]. Briefly, the grape seed powder of 0.100 g was thoroughly mixed with 1 mL of the tannin extraction buffer (70% acetone/water and 0.1% acetic acid) and treated ultrasonically for 30 min in an ice-water bath in the dark. The supernatant containing soluble CT were collected by centrifugation for 10 min at 4 °C and 12,000 rpm, and the remaining sediment was used for the analysis of insoluble CT.

The supernatant containing soluble CT was further extracted successively with equal volumes of chloroform and hexane to completely remove the lipid. The lower solution containing the soluble CT was then lyophilized for 24 h, and the dry matter obtained was redissolved in 500 µL of 50% methanol/water solution. The absorbance value of the soluble CT reaction with 1% 4-(dimethylamino) cinnamaldehyde (DMACA) solution (*w*/*v*) dissolved in 1:1 methanol/6 mol/L; the hydrochloric acid was determined at 640 nm on the microplate reader (SpectraMax 190, Molecular Devices, San Jose, CA, USA), as described in our publication [30]. The soluble CT were quantified relative to the standard curve generated from the (−)-epicatechin concentration and the corresponding absorbance values.

To determine the concentration of insoluble CT, the insoluble CT sediment obtained above was lyophilized. The dried powder containing insoluble CT was then completely mixed with 1 mL of the butanol/HCL (95:5, *v*/*v*) solution, vortexed, sonicated for 30 min in the dark, and incubated in a metal bath at 95 °C for 1 h. The absorbance of this reaction was measured at 550 nm on the microplate reader, as described previously [30]. The insoluble CT were quantified relative to the standard curve generated from the procyanidin B1 concentration and the corresponding absorbance values.

#### 4.2.4. Determination of Flavan-3-ol Units

The constituent units of soluble and insoluble CT were analyzed following the method described in the publication [30]. Briefly, soluble and insoluble CT, respectively, were split into constituent units using an excess amount of phloroglucinol lysate consisting of 0.5% ascorbic acid, 0.3 mol/L hydrochloric acid, and 50 g/L phloroglucinol/methanol solution. The lysate solution was lyophilized for 24 h and the sediment was redissolved in 200 μL of a 50% chromatography-grade methanol/water solution. The extension unit with phloroglucinol and the terminal unit without phloroglucinol were detected using an Agilent 1200 series high-performance liquid chromatography triple quadrupole mass spectrometer (HPLC-QqQ-MS/MS). The analytical parameters of the instrument were set, as described by Liu et al. [30].

As the soluble CT extraction contained free flavan-3-ol monomers, the free flavan-3-ols in seeds were calculated separately. The free flavan-3-ol monomers and terminal units were quantified using the (+)–catechin, (−)-epicatechin, and (−)-epicatechin-3-*O*-gallate standard curves, and the extension units were quantified using the standard curve of the standard PB1 after lysis under the same conditions as the sample. The concentration of the flavan-3-ol units was expressed in mg per g seed. The mDP of soluble and insoluble CT, respectively, was estimated using the following equations”

(2) $$\mathrm{mDP}\;(\mathrm{Soluble}\;\mathrm{CT})=\;\frac{\mathrm{Extension}\;\mathrm{units}+\mathrm{Free}\;\mathrm{monomers}\;\mathrm{after}\;\mathrm{lysis}-\mathrm{Free}\;\mathrm{monomers}\;\mathrm{before}\;\mathrm{lysis}}{\mathrm{Free}\;\mathrm{monomers}\;\mathrm{after}\;\mathrm{lysis}-\mathrm{Free}\;\mathrm{monomers}\;\mathrm{before}\;\mathrm{lysis}}\;\mathrm{mDP}\;(\mathrm{Insoluble}\;\mathrm{CT})=\;\frac{\mathrm{Terminal}\;\mathrm{units}+\mathrm{Extension}\;\mathrm{units}}{\mathrm{Terminal}\;\mathrm{units}}$$

Note: “Free monomers after lysis” refers to the sum of flavan-3-ol monomer concentration and terminal units, and “free monomers before lysis” refers to the concentration of flavan-3-ol monomers.

### 4.3. Statistical Analysis

The data for each point in these graphs are the mean values of three biological plot samples (*n* = 3). Two-way ANOVA (Duncan’s, *p* < 0.05) and independent samples t-test were performed using SPSS Statistics 26 (IBM Corporation, Armonk, NY, USA). OriginPro 2022 (OriginLab, Northampton, MA, USA) was used for correlation analysis, and all plots were completed using GraphPad Prism 8.0.2 (GraphPad Software, San Diego, CA, USA). Based on the determined CIELAB chromaticity index, the color of the seed coat was reproduced using Adobe Photoshop CS6 13.0 (Adobe Systems, Inc., San José, CA, USA).

## 5. Conclusions

In summary, this study investigated the changes in soluble and insoluble CT in the ‘Cabernet Sauvignon’ grape seeds from two vineyards over two years. It is suggested that a lower sugar increment in the developing grape berries favors the conversion of seed CT towards a higher degree of polymerization. In wine grape production, the seeds should be fully ripened to reduce the bitter galloylation component of tannins. In addition, this study also found that the seed coat color can predict to some extent the polymerization of CT. The results provide a reference for the control of the grape berry and seed ripening process for the production of high-quality wine.

## Figures and Tables

**Table 1:** Two-way ANOVA for effects of year and plot on soluble and insoluble CT content in seeds.

CT Type	Year	Plot	Year × Plot
Soluble CT	***	ns	***
Insoluble CT	*	***	ns

The asterisk indicates that there is a statistical difference between the 2 vineyards in the same period at the 0.05 level: *, *p* < 0.05; ***, *p* < 0.001; ns, not significant.

**Table 2:** Sampling schedule in 2019 and 2020.

Year	Date of Grape Sampling and Days after Full Flowering							
2019	25 July	6 August	17 August	26 August	5 September	16 September	25 September	8 October
	63	75	86	95	104	116	125	138
2020	19 July	27 July	8 August	19 August	28 August	7 September	17 September	26 September
	55	63	75	86	94	104	114	123

## Data Availability

The data used for the analysis in this study are available within the article.
